# Influence of endurance and endurance–strength training on mineral status in women with abdominal obesity: a randomized trial

**DOI:** 10.1097/MD.0000000000014909

**Published:** 2019-03-22

**Authors:** Damian Skrypnik, Paweł Bogdański, Katarzyna Skrypnik, Edyta Mądry, Joanna Karolkiewicz, Monika Szulińska, Joanna Suliburska, Jarosław Walkowiak

**Affiliations:** a Department of Treatment of Obesity, Metabolic Disorders and Clinical Dietetics, Poznan University of Medical Sciences; b Institute of Human Nutrition and Dietetics, Poznan University of Life Sciences; c Department of Physiology, Poznan University of Medical Sciences; d Department of Physiology, Biochemistry and Hygiene, Poznan University School of Physical Education; e Department of Pediatric Gastroenterology and Metabolic Diseases, Poznan University of Medical Sciences, Poznan, Poland.

**Keywords:** mineral status, obesity, physical activity

## Abstract

**Background:**

Obesity and exercise are associated with disturbances of mineral metabolism, which can lead to physical inefficiency. Our study aimed to compare the influence of endurance and endurance–strength training on mineral status in women with abdominal obesity.

**Methods:**

Thirty-eight abdominally obese women were randomized into groups A and B and underwent 3 months long training: group A—endurance training and group B—endurance–strength training. Anthropometric and body composition measurements were carried out and the Graded Exercise Test was performed. Blood, urine, and hair samples were collected for mineral content analysis.

**Results:**

Endurance training decreased serum Fe and Zn concentrations as well as hair Zn and Cu content, and increased urine Zn concentration. Endurance–strength training increased serum Mg and Cu concentrations, decreased serum Fe and Zn concentrations, decreased hair Ca and Mg content, and increased urine Ca and Zn concentrations. After training, serum and urine Fe concentration was higher in group A, while urine Ca concentration was higher in group B. A number of correlations was found.

**Conclusions:**

Both endurance and endurance–strength training have a significant effect on mineral metabolism in obese women; the favorable effects of endurance–strength exercise predominate in iron, magnesium, zinc, and copper balance.

## 1 Introduction

Overweight and obesity are associated with a wide range of mineral metabolism disorders, in both causal and consequential modes.^[1–4]^ It has been shown that dietary calcium (Ca) intake is inversely correlated with nutritional indicators such as fat mass index,^[5]^ body mass index (BMI),^[6]^ subscapular skinfold thickness,^[7]^ central obesity in Caucasian women,^[8]^ and the constituents of metabolic syndrome independent of ethnicity, gender, and lifestyle factors.^[1]^ These associations appear to be significantly stronger in women than in men.^[9]^ Excess body mass causes serious disruption of cellular cytosolic and organelle calcium homeostasis, especially in metabolically active tissues, such as hepatic and fat tissue. These calcium metabolism alterations lead to increased hepatic glucose production, lipogenesis, and inflammation.^[2]^

There have been a number of demonstrations of the bilateral dependence between iron (Fe) metabolism and obesity. It has been shown that serum ferritin concentration is positively correlated with BMI,^[10]^ visceral fat area, subcutaneous fat area, hepatic fat content, and homeostasis model assessment (HOMA),^[11]^ which makes ferritin an indirect marker of insulin resistance. Overweight and obesity are frequent causes of iron deficiency or, inversely, of iron overload.^[4]^

Magnesium (Mg), zinc (Zn), and copper (Cu) metabolism disorders are also tightly linked to excess body mass. A strong association has been observed between low serum magnesium concentration and obesity.^[12]^ It has been demonstrated that low zinc blood concentration is associated with dyslipidemia, inflammatory state, and insulin resistance in patients with excess body mass.^[13]^

Blood copper level has been seen to be elevated in humans with obesity,^[14]^ though this has not been confirmed by all studies.^[15]^ Reduced hepatic copper levels are found in human nonalcoholic fatty liver disease (NAFLD), which is a hepatic manifestation of metabolic syndrome.^[4,16]^

The American Heart Association recommends physical exercise in obesity as it helps reduce cardiometabolic risk factors, such as metabolic syndrome, cardiovascular disease, insulin resistance and type-2 diabetes, elevated blood pressure, dyslipidemia, and inflammation.^[17]^ Exercise significantly affects mineral metabolism. Intense aerobic training significantly reduces Ca^2+^ cellular amplitude and impairs myocardial Ca handling, leading to cardiomyocyte dysfunction in both the left and right ventricles.^[18]^ Endurance training, on the contrary, exerts an antiarrhythmic effect after myocardial infarction. Moreover, this type of training normalizes calcium homeostasis in the intracellular fluid of myocytes.^[19]^ Physical training modulates the expression of mitochondrial calcium uniporter, which is responsible for mitochondrial calcium homeostasis.^[20]^ On the other hand, a high pre-exercise calcium dietary supply has been shown to reduce calcium abnormalities in bone tissue among female endurance cyclists.^[21]^ High-intensity short-term mixed endurance–strength training exerts no direct effect on serum iron; however, after a recovery period, the serum iron level significantly decreases. Unlike in the case of iron, mixed endurance–strength training results in an instantaneous increase in serum ferritin and hepcidin levels, which normalize after a 24-h rest.^[22]^ Despite a number of uncertainties, it has been demonstrated that undisturbed magnesium metabolism and magnesium supplementation are tightly associated with increased exercise performance, as measured by muscle strength and runtest outcomes. Particularly, improvements in muscle strength parameters in the elderly^[23]^ and the amelioration of gait speed and chairstand times in elderly women who have undergone an exercise program with supplemental magnesium intake have been documented.^[24]^ These findings show that the presence of the correct magnesium level during exercise has a protective effect against degenerative lean muscle loss.^[23]^ Results of studies on the effect of exercise on zinc metabolism are strongly inconsistent, providing no clear conclusion.^[25,26]^ Aerobic endurance and strength exercise have a direct increase on serum copper levels,^[26,27]^ despite the significant copper loss in the urine,^[28]^ which cannot be fully compensated for after recovery.^[29]^ There is clearly a lack of current human studies of the effect of training on zinc and copper metabolism.

The aim of this study was to compare the influence of endurance and endurance–strength training on the mineral status of calcium, iron, magnesium, zinc, and copper in women with abdominal obesity. As mentioned above, both obesity and physical exercise significantly modify microelement and macroelement metabolism; however, the effect of these 2 factors on mineral balance has to date been examined separately. To the best of our knowledge, ours is the first study worldwide on the effect of exercise on mineral alterations in a population with excess body mass. So far, the effect of the coincidence of these 2 states on mineral status has not been investigated. Moreover, the uniqueness of our study arises from the fact that, in the trial, 2 different modalities of training were compared.

## 2 Materials and methods

### 2.1 Study patients

Informed consent in writing was obtained from each subject. The study protocol was approved by the Ethics Committee, Poznań University of Medical Sciences (case no. 1077/12 with supplement no. 753/13). The trial meets the standards of the Declaration of Helsinki (1975 revision with amendments). One hundred and sixty-three obese female patients from the Department of Internal Medicine, Metabolic Disorders, and Hypertension at Poznań University of Medical Sciences, Poland were screened and a total of 44 were enrolled. The study has been registered on ClinicalTrials.gov under the ID NCT03444207. The study protocol can be accessed at https://clinicaltrials.gov/ct2/show/NCT03444207.

The inclusion criteria were written informed consent; obesity defined as BMI ≥30 kg/m^2^; age 18–65 years; content of body fat assessed by electrical bioimpedance ≥33%; waist circumference >80 cm; and stable body weight in the month before enrollment.

The exclusion criteria were any secondary form of obesity or secondary form of arterial hypertension; mean systolic blood pressure greater than 140 mm Hg, mean diastolic blood pressure greater than 90 mm Hg; coronary artery disease; stroke; heart failure; diabetes; malignancy; disturbances of heart rhythm; use of dietary supplements; serious liver or kidney abnormalities; disrupted thyroid gland function; connective tissue disease, inflammation, or arthritis; alcohol, nicotine, or narcotic abuse; pregnancy, childbirth, or lactation; acute or chronic infection, as well as any other condition that would make participation not in the best interest of the subject, or could prevent the efficacy of the study. Occurrence of any of the exclusion criteria during the study resulted in the subject being withdrawn from the study. Detailed inclusion and exclusion criteria are described in our previous studies.^[16,30,31]^

### 2.2 Study design

The study was performed as a prospective randomized trial. Subjects were enrolled by physician and randomized into group A and group B using a randomization list. Both groups underwent physical training of 3 months’ duration. Group A performed endurance training while group B performed endurance–strength training. Subjects were asked to maintain the level of physical activity and diet they were used to. At baseline, and after the 3 months of training, blood samples, urine samples, and hair samples were taken for laboratory analysis, and anthropometric measurements, body composition measurements, and Graded Exercise Test (GXT) were performed for both groups. The detailed study design and GXT procedures are presented in our previous studies.^[16,30,31]^ The data were collected in the metabolic laboratories of Department of Treatment of Obesity, Metabolic Disorders and Clinical Dietetics, Poznan University of Medical Sciences and Department of Physiology, Biochemistry and Hygiene, Poznan University School of Physical Education. Mineral analysis was performed in the laboratory of Institute of Human Nutrition and Dietetics, Poznan University of Life Sciences.

### 2.3 Anthropometric measurements

Anthropometric measurements were performed in the morning, under metabolic laboratory conditions, after a night's rest and with the subjects wearing light clothing and no shoes. Height was measured to the nearest 0.5 cm and weight to the nearest 0.1 kg. BMI was calculated as weight divided by height squared (kg/m^2^). Waist circumference (cm) and hip circumference was measured to the nearest 0.5 cm.^[30,32]^

### 2.4 Body composition measurements

Body composition analysis was assessed by dual energy X-ray absorptiometry (DXA) (GE Healthcare Lunar Prodigy Advance, GE Medical Systems, Milan, Italy), which is said to be the gold standard for body composition measurements in obese patients. Subjects were instructed to avoid excess physical effort in the 24 h before the analysis. The DXA measurement was performed in the morning, after a night's rest, fasting. Subjects were instructed in detail regarding the measurement procedure. During the procedure, patients wore cotton T-shirts, shorts, and socks with no metal, rubber, and plastic objects, and laid on the DXA table supine and motionlessly. The same trained operator positioned the subjects, performed the DXA scans, and executed the analysis according to the operator's manual, using the standard analysis protocol. The standard scan mode (for moderately obese subjects) or the thick scan mode (for extremely obese subjects), with absorbed radiation doses of 0.4 μGy and 0.8 μGy, respectively, were used. The intrasubject and intersubject coefficients of variation (CV% = 100 × standard deviations [SD]/mean) ranged from 1% to 5%. The coefficient of variation for the bone mass measurements was <1%. Anthropometric and DXA measurements are described in detail in our previous studies.^[16,30,31]^

### 2.5 Intervention

The training program lasted 3 months, with 3 sessions weekly. A total of 36 training sessions took place in both study groups. Subjects in group A performed endurance exercise on cycle ergometers (Schwinn Evolution, Schwinn Bicycle Company, Boulder, CO). Group B underwent endurance–strength training. The strength component consisted of exercises with a neck barbell and a gymnastic ball. Directly after the strength exercise, the subjects underwent the endurance component with cycle ergometers (Schwinn Evolution). Both the endurance and endurance–strength training were comparable in exercise volume; the only difference was the modality of the effort. In both groups, each training session lasted an hour. Training was supervised by a certified instructor in professional gymnastic, and medical supervision was provided. The training program is described in detail in our previous studies.^[16,30,31]^

Both endurance and endurance–strength training resulted in significant decreases in body mass, BMI, waist circumference, hip circumference, and total body fat.^[30]^ Only in group B was a significant increase in total body lean mass observed.^[30]^ Both endurance and endurance–strength training led to increases in exercise maximal work rate and exercise peak oxygen uptake.^[30]^ After the study, no significant differences were found in anthropometric parameters, body composition parameters, or results of GXT analysis^[30]^ between the study groups. Detailed results of the anthropometric, body composition, and GXT analysis are presented in our previous studies.^[30]^

### 2.6 Collection of blood, urine, and hair samples

At baseline and at the completion of the 3-month intervention, fasting blood samples from the forearm vein, the first morning urine samples, and hair samples were taken. A 1-cm-long hair strand was collected from the occiput scalp of each subject after the hair had been washed with a shampoo containing no functional components, and put into separate labeled paper bags. Patients were instructed not to dye their hair or use hair spray during the study. Hair samples were washed in acetone and deionized water, before being dried at 105–110°C. They were then dried and weighed. Hair that was permed or dyed was not used.

### 2.7 Mineral content measurement

The calcium, iron, magnesium, zinc, and copper contents of the serum, urine and hair were determined after digestion in 65% (w/w) spectra pure HNO_3_ (Merck, Kenilworth, NJ) in the Microwave Digestion system (Mars 5, CEM, Matthews, NC). After digestion and dilution with deionized water and LaCl_3_·7H_2_O (Ca, Mg), the concentrations of calcium, iron, magnesium, zinc, and copper in the mineral solutions were determined using flame atomic absorption spectrometry (AAS-3, Carl Zeiss, Jena, Germany). The mineral contents of the serum, urine, and hair were measured at wavelengths of 422.7 nm for calcium, 285.2 nm for magnesium, 213.9 nm for zinc, 248.3 nm for iron, and 324.8 nm for copper. The accuracy of the method was verified with certified reference materials (HUM ASY CONTROL 2 and URN ASY CONTROL 2, Sero, Billingstad, Norway and Human Hair NCS DC73347a, LGC, Teddington, UK) and was 93% to 96% for calcium, 99% to 102% for magnesium, 95% to 96% for zinc, 95% to 98% for iron, and 99% to 103% for copper. The procedure for determining mineral levels is described in our previous study.^[33]^

No changes to trial outcomes after the trial commenced were implemented.

### 2.8 Dietary and supplement intake

Dietary intake was determined using dietary intake interviews at baseline, every 14 days during the study intervention, and completion of the trial. Qualitative and quantitative analysis of the diet was performed using a dietetics computer program. All subjects were asked not to use dietary supplements. The total caloric intake, mineral intake (Ca, Fe, Mg, Zn, Cu in this range), nutrient intake, caffeine consumption, nutrition mode, and eating habits were comparable between the groups, and stable and constant during whole intervention. The dietary analysis is described in our previous publications.^[16,30,31]^

### 2.9 Randomization and masking and statistical analysis

All included patients were given a unique code as an identifier. The random allocation sequence with the use of the list of patients’ unique codes was computer-generated. It was impossible for the study personnel involved with the patients to adjust the randomization. Patients were assigned to intervention by physician with the use of patient's unique code according to random allocation sequence generated by computer.

Data are presented as means ± SDs. All statistics and calculations were performed using Statistica 10.0 software (StatSoft, 1984–2011, Kraków, Poland). The Shapiro–Wilk test was used to check the normal distribution. Comparisons between groups were carried out using the unpaired *t* test (for data with normal distribution) or the Mann–Whitney *U* test. The paired *t* test (for data with normal distribution) or the Wilcoxon rank–sum test was used to determine the statistical significance of the variables before and after intervention. The Pearson correlation test (for data with normal distribution) or Spearman's rank analysis was performed to calculate correlation coefficients. A *P* value of less than .05 was regarded as significant. It was calculated that a sample size of at least 16 subjects in each group would yield at least 80% power of detecting an intervention effect that was statistically significant at the .05 α level.

## 3 Results

A total of 44 subjects from the 163 screened at the outpatient clinic of Department of Internal Medicine, Metabolic Disorders, and Hypertension at Poznań University of Medical Sciences fulfilled all the inclusion criteria and none of the exclusion criteria. These women were randomized into groups A and B of 22 women each. During the intervention, 6 patients (1 from group A and 5 from group B) were excluded due to poor compliance. Thirty-eight women (21 from group A and 17 from group B) completed the trial and underwent statistical analysis. The analysis was performed by original assigned groups. The recruitment and follow-up period lasted from December 2012 to May 2013. The trial ended when the intervention of the last subject was completed. No important harms or unintended effects occurred. The baseline characteristics of the study groups are presented in Table 1.^[30]^ The subjects’ compliance ratio was 86.4%.^[30]^ There were no differences at enrolment in anthropometric parameters, body composition parameters, or GXT analysis between study groups. No important changes to methods after trial commencement have been implemented.

**Table 1:**
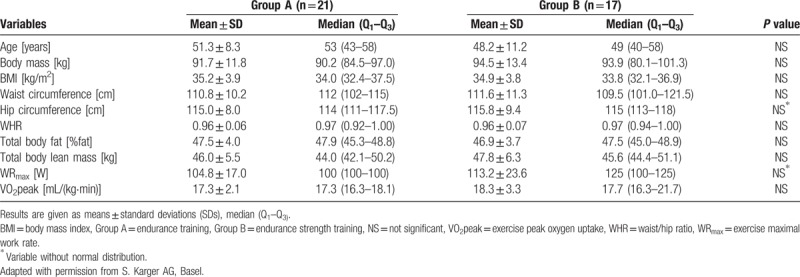
Baseline characteristics of study groups^[30]^.

Before training, there were no significant differences in serum, hair, and urine content of calcium, iron, magnesium, zinc, and copper between the study groups. In group A, endurance training led to a significant decrease in serum Fe and Zn concentration, a decrease in hair Zn and Cu content, and an increase in urine Zn concentration. In group B, endurance–strength training led to significant increases in serum Mg and Cu concentration, significant decreases in serum Fe and Zn concentration, a decrease in hair Ca and Mg content, and an increase in urine Ca and Zn concentration. After the training, serum and urine Fe concentrations were significantly higher in group A than in group B, and urine Ca concentration was significantly higher in group B than in group A. The changes observed in the levels of the analyzed minerals before and after the training, and a comparison between groups, are presented in Table 2. Comparing changes in analyzed mineral parameters between group A and B significant differences were found in changes of hair Mg and Zn content. Comparison of changes in mineral parameters between group A and B is presented in Table 3. A number of significant correlations between mineral parameters and anthropometric, body composition, and GXT parameters were found. These correlations are shown in Table 4.

**Table 2:**
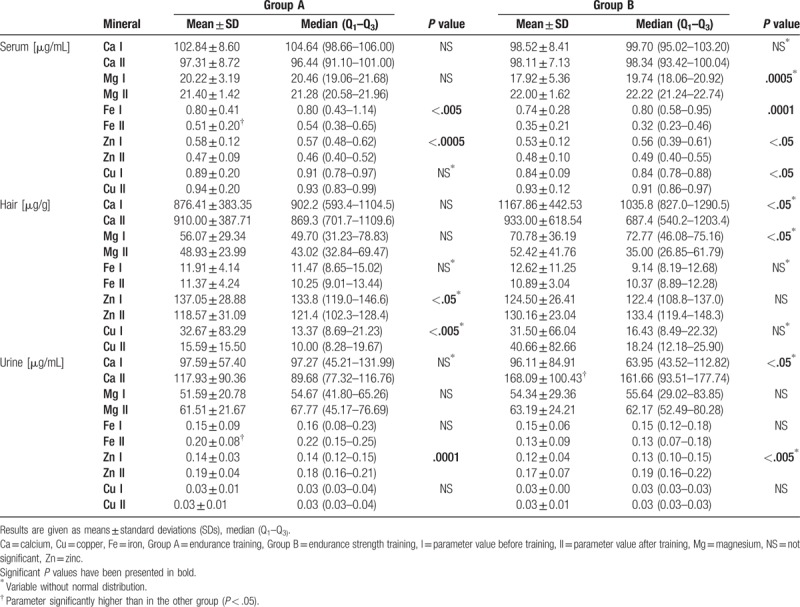
Mineral content before and after training.

**Table 3:**
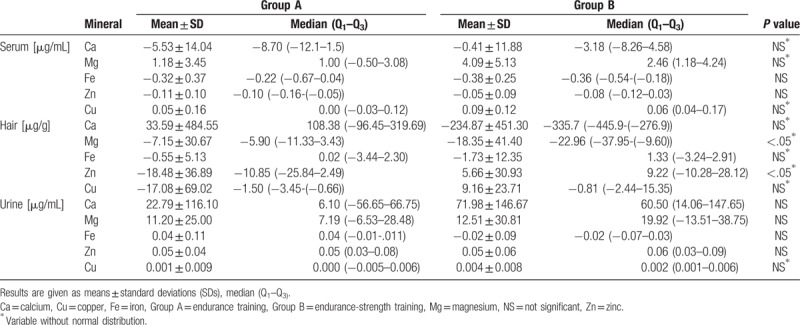
Comparison of changes (Δ) in mineral parameters between group A and B.

**Table 4:**
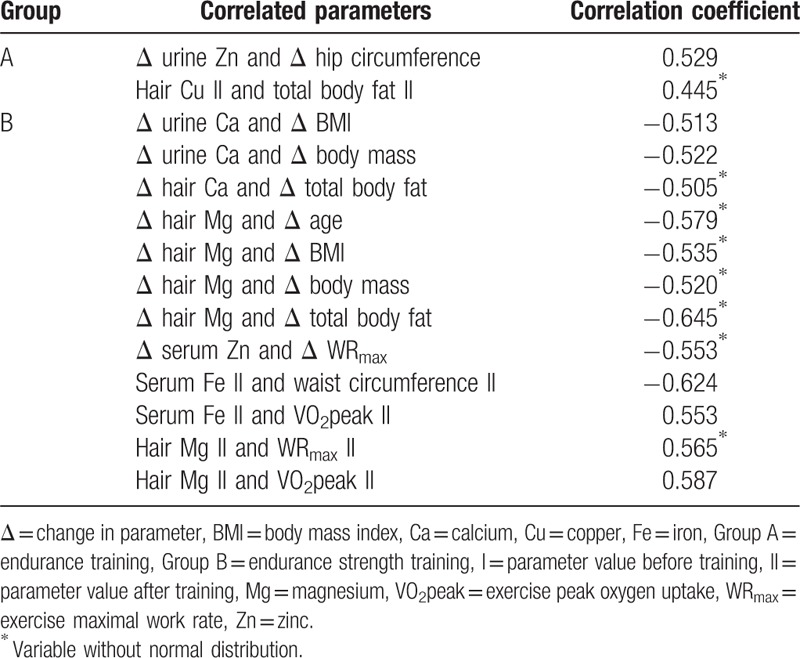
Significant (*P* < .05) correlations of mineral content and examined parameters.

## 4 Discussion

Our study has shown significant changes in calcium, iron, magnesium, zinc, and copper contents of serum, hair, and urine as a result of 2 different modes of short-term physical training in abdominally obese women. The main differences between analyzed types of exercise were higher serum and urine Fe concentration and lower urine Ca concentration after endurance training, compared to endurance–strength training. These are the first findings of this type ever reported.

Calcium metabolism disturbances and decreased bone mineral density (BMD) in female professional cycling athletes are well documented. The main reason for this is low energy intake and the non-weight-bearing nature of this sport in professional sportswomen.^[21]^ In our trial on obese women who were not professional athletes, no Ca alterations were observed in group A, in contrast to group B, where calciuria increased significantly after training, as compared to group A; Ca hair content also decreased, reflecting lower Ca body deposits; however, with no differences in changes in Ca parameters between groups. This raises the hypothesis that excess body mass plays a protective role against calcium loss in pure endurance cycling exercise. Moreover, these observations show that pure endurance cycling training, unlike endurance cycling training with an additional strength component, does not have a negative effect on calcium balance. Thus, taking into consideration the fact that physical inactivity is a risk factor of postmenopausal osteoporosis and osteoporotic fractures,^[34]^ endurance exercise can be recommended as a prevention strategy for women at risk of such disturbances. In group B, though not in group A, the decrease in body mass and BMI correlated negatively with the increase in calciuria. Given that a negative correlation was observed between the decrease in total body fat and the decrease in hair Ca content in group B, it may be the case that fat tissue is at least in part responsible for the disturbances in Ca metabolism observed in obesity. This association has been previously pointed out by Lind et al.^[35]^

In our study, we saw a decreased serum iron concentration after both endurance and endurance–strength training, though with no accompanying increase in iron loss in the urine, and with no differences in changes in Fe parameters between groups. It has been shown that athletic exercise increases bone marrow iron uptake and enhances erythropoiesis.^[36]^ Interestingly, serum Fe concentration in group B after the training was positively correlated with exercise peak oxygen uptake, which depends on the hemoglobin concentration in the blood;^[30]^ this shows that the endurance–strength exercise led to improvements in the process of marrow intake of iron and hemoglobin synthesis. We thus hypothesize that both short-term endurance and endurance–strength exercise lead iron to shift to the bone marrow in obesity; however, this effect is strongly following endurance–strength physical effort, on account of the lower iron serum content after training in group B than in group A. The predominance of the effect of mixed endurance–strength training on Fe metabolism also arises from the higher iron loss in the urine after exercise in group A, as compared to B. We documented an inverse correlation in group B between serum iron concentration and waist circumference following training. This suggests that there is a close relationship between iron deficiency and the abdominal type of obesity.

The study of Bertinato et al^[12]^ demonstrated a link between low serum magnesium concentrations and excess body mass, insulin resistance, and diabetes in women—but not in men. Our study confirmed an association between magnesium metabolism and anthropometric parameters—decreases in body mass, BMI, and total body fat correlated negatively with decreases in hair Mg, though only in group B. Our study also demonstrated that endurance–strength training in obese patients affects Mg homeostasis, contrary to endurance training alone. In group B, a shift of magnesium from tissues to blood resulted from increased Mg serum concentration, while the decrease in Mg hair content was stronger in older patients, without concomitant changes in magnesiuria after training. Mg is crucial to energy metabolism, cardiorespiratory function, muscle action, and high exercise performance.^[23]^ Our study is in line with these findings, in the case of obese women who underwent endurance–strength training: a positive correlation was found in group B between hair Mg and exercise maximal work rate and exercise peak oxygen uptake after training.^[30]^ Studies have shown changes in serum zinc levels following exercise: increases in the case of endurance training^[25]^ and decreases with strength exercise.^[26]^ Serum Zn decrease is accompanied by increased zincuria following strength training.^[26]^ In our study, zinc excretion intensified, as shown by the decreased serum Zn concentration and increased urine Zn level, independently of training mode. However, in group B, an increase in exercise maximal work rate correlated negatively with a decrease in serum Zn level. Moreover, in group A, the decrease in hip circumference correlated positively with the increase in urine Zn concentration. Additionally, in group A—unlike in group B—Zn loss was observed in the hair. Thus, elevated body mass seems to be a factor that is responsible at least in part for the response of Zn metabolism to endurance exercise. Poor serum zinc content in obesity is a risk factor for hypercholesterolemia, diabetes, and increased inflammation.^[13]^ Thus, preventing zinc disturbances in patients with excess body mass undergoing training is crucial.

Both animal and human studies have shown that copper deficiency induces a range of metabolic syndrome components, such as insulin resistance,^[37]^ atherogenic dyslipidemia,^[38]^ hypertension, increased cholesterol and triglycerides, and altered lipoprotein composition.^[37]^ A recent very interesting study of Tarantino et al^[39]^ on 100 obese patients with low prevalence of comorbidities has demonstrated the negative prediction of intima-media thickness by the copper serum level. This finding emphasize the tight connection between copper bioavailability, early stages of atherosclerosis and cardiovascular risk in patients with excess body mass. Tarantino et al^[39]^ results refer mainly to patients with hepatic steatosis, a hepatic manifestation of metabolic syndrome.^[16]^ These findings allow to hypothesize that proper Cu metabolism may act as 1 of key factors in NAFLD prevention and cardiovascular risk management. In our study, an increase in serum Cu concentration following endurance–strength training and a decrease in Cu hair content after pure endurance training were observed. Moreover, in group A after training, the hair Cu content correlated positively with fat body content. These findings suggest that there is a connection between fat tissue and Cu homeostasis. We thus hypothesize that the 2 types of training investigated here have contrary effects on copper metabolism response in obesity, with the predominant favorable effect coming from mixed training. However, in our study no differences in changes in Cu parameters between groups were registered, probably due to size of the study group not large enough to show statistical significance.

### 4.1 Limitations

The main limitation of the trial was the relatively small size of the study group. The main reason for this was the rigorous inclusion and exclusion criteria, which did however enable us to eliminate a large number of disrupting factors. In addition, to investigate iron metabolism in detail, a range of parameters such as ferritin, transferin, total iron-binding capacity, and unsaturated iron-binding capacity should have been used. Similarly, to investigate calcium metabolism in detail, some additional parameters like parathyroid hormone, vitamin D, and procollagen I amino-terminal propetide should have been examined. However, these could not be obtained by the mineralization technique used here.

### 4.2 Strengths

To eliminate the influence of gender, only women were included in the trial. This was due to data on the differing response to exercise in women and men ^[40]^ and because stronger mineral balance abnormalities are observed in females. Moreover, the prevalence of obesity is greater in women compared to men. Also the high compliance ratio (over 85%), the elimination of dietary influence, the comparative mode, and the analysis not of serum and urine, but also of hair, are all strong points of this study.

## 5 Conclusions

Both endurance and endurance–strength training exert significant effects on calcium, iron, magnesium, zinc, and copper metabolism in obese women, with the predominance of favorable effects on iron, magnesium, zinc, and copper balance coming from mixed endurance–strength exercise. Further large-scale studies are necessary to determine the modality of training that most favorably ameliorates mineral status in obesity.

## Acknowledgments

We acknowledge Agnieszka Seraszek, PhD (Department of Bioinformatics and Computational Biology; Poznan University of Medical Sciences) for statistical consultation. Agnieszka Seraszek, PhD has given permission to be named.

## Author contributions

Conceptualization, J.W., P.B., D.S., J.S., and K.S.; methodology, J.W., P.B., J.K., D.S., and J.S.; software, J.S., K.S., E.M., and M.Sz.; validation, J.S., K.S., E.M., and M.Sz.; formal analysis, D.S., K.S., J.S., and P.B.; investigation, D.S., K.S., J.K., J.S., and P.B.; resources, D.S., P.B., K.S., and M.Sz.; data curation, D.S., P.B., J.W., and J.S.; writing—original draft preparation, D.S. and K.S.; writing—review and editing, J.S., J.W., P.B., J.K., E.M., and M.Sz.; visualization, E.M., M.Sz., D.S., and K.S.; supervision, J.W., P.B., J.S., and J.K.; project administration, J.W., P.B., J.K., J.S., and D.S.; funding acquisition, J.W., P.B., J.S., and J.K.

Damian Skrypnik orcid: 0000-0001-5643-6899.
